# Pancancer analysis of oncogenic BARX2 identifying its prognostic value and immunological function in liver hepatocellular carcinoma

**DOI:** 10.1038/s41598-023-34519-8

**Published:** 2023-05-09

**Authors:** Shian Yu, Yu Yang, Hanqing Yang, Long Peng, Zhipeng Wu, Liang Sun, Zhengyi Wu, Xuzhe Yu, Xiangbao Yin

**Affiliations:** 1 Department of Hepatobiliary Surgery, The Second Affiliated Hospital of Nanchang University, Nanchang, 330006 China; 2 Department of Neurosurgery, Affiliated Hospital of Nantong University, Nantong, 226001 China

**Keywords:** Computational biology and bioinformatics, Immunology, Oncology

## Abstract

The transcription factor BarH-like homeobox 2 (BARX2), a member of the Bar-like homeobox gene family, is involved in cell proliferation, differentiation, immune responses and tumorigenesis. However, the potential role of BARX2 in the development of liver hepatocellular carcinoma (LIHC) remains unclear. Therefore, we aimed to study the biological role of BARX2 in hepatocellular carcinoma. Through the UALCAN, GTEx PORTAL, TIMER 2.0, LinkedOmics, SMART, MethSurv, Metascape, GSEA and STRING public databases, the BARX2 mRNA level, prognostic value, coexpressed genes, associated differentially expressed genes, DNA methylation and functional enrichment of LIHC patients were studied. The relationships between BARX2 expression and various clinical or genetic parameters of LIHC patients were determined using data from The Cancer Genome Atlas (TCGA), Gene Expression Omnibus (GEO), and BEAT LIHC databases. In addition, the biological function of BARX2 in LIHC was studied in vitro. Through large-scale data mining, our study showed that BARX2 was differentially expressed between different normal and tumour tissues.BARX2 expression in LIHC tissues was significantly lower than that in corresponding controls, especially in patients with T2-4 stage disease. In patients with LIHC, overexpression of BARX2 was an independent poor prognostic factor associated with poor cytogenetic risk and gene mutations. Genomic hypermethylation of the BARX2 gene was associated with upregulated BARX2 expression and poor overall survival (OS) in LIHC. Functional enrichment analysis showed that BARX2 had an immunomodulatory role and was involved in the inflammatory response in LIHC occurrence. In conclusion, the oncogene BARX2 may serve as a new biomarker and prognostic factor for patients with LIHC. The immunomodulatory function of BARX2 deserves further validation in LIHC.

## Introduction

Liver cancer is a global public health problem, with an annually increasing incidence rate^1^. While hepatocellular carcinoma is one of the major types of liver cancer, it is also the second most malignant disease causing cancer-related death worldwide^2^. WHO surveys show that by 2025, approximately one million people will be affected by liver cancer each year^3^. Due to the early dissemination of LIHC to the liver, this disease is usually multinodular at clinical diagnosis and LIHC tumour cells have a marked affinity for intravascular growth and can invade the portal vein or hepatic veins^4^. In recent years, tumour immunology has received increasing attention in the context of LIHC pathogenesis, and studies have shown that immunosuppressive molecules are effective in hepatocellular carcinoma^5–8^. In addition, some scientists have demonstrated that by targeting the signalling pathways of tumour angiogenesis and tumour proliferation, the survival time of patients can be prolonged; in summary, tumour immunotherapy has reshaped the clinical treatment prospects of hepatocellular carcinoma^9,10^.

BARX (BarH-like homeobox) is a class of transcriptional regulators encoding transcription factors containing homologous structural domains, and is named because of its similarity to the Bar subclass of homologous structural domain proteins involved in Drosophila eye development^11^. To date, two members of the BARX protein family have been identified in mammals, named BARX1 and BARX2 for the order in which they were discovered^12^. They are widely expressed in a variety of tissues; perform diverse biological functions in cell proliferation, differentiation, growth factor signalling, transcriptional regulation and embryonic development; are key regulators of the respiratory, circulatory, digestive and immune systems; and are implicated in inflammation, cancer and other diseases^13–16^. BARX2 was discovered in 1996 by Jones et al. in the process of screening a mouse embryo expression library, and this new homologous cassette gene is similar to Barx1 and the Drosophila Bar gene, hence the name Barx2^13^. BARX2, also known as barh-like homodimer 2, is located at 11q24-q25 and encodes a homologous transcription factor consisting of 254 amino acids^17^. The gene encoding human Barx2 maps to chromosome 1, 1q25, with four exons ranging from 85 to 1099 bp^18^. BARX2, a homologous structural domain factor of the Bar family, regulates factors that control the expression of cell adhesion molecules and affects cell differentiation in various developmental contexts^13–16,19–21^. Dysregulation of BARX2 and its oncogenic role in the development of various tumours such as ovarian, gastric and lung cancers have been reported^22–24^. However, the role of BARX2 in hepatocellular carcinoma is very poorly understood. Here, we conducted an in-depth study on the expression of BARX2 in hepatocellular carcinoma and its prognostic value. Furthermore, the clinical significance, potential molecular functions and regulatory networks of BARX2 in LIHC patients were investigated by bioinformatic analysis of datasets available from public databases.

## Results

### Tissue-specific expression of BARX2 in a pancancer dataset

**Figure 1:**
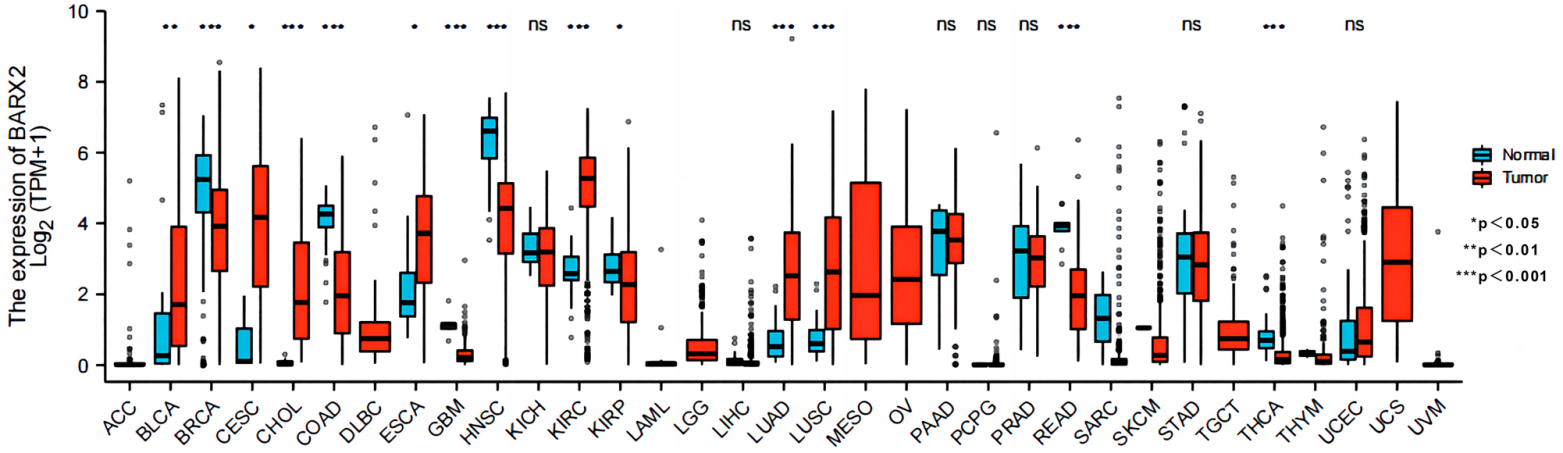
Differential expression of BARX2 in cancerous and normal tissues in the TCGA and GTEx databases was analysed by the Mann‒Whitney U test. ***P < 0.001, **P < 0.01, *P < 0.05. *LIHC* liver hepatocellular carcinoma, *ACC* adrenocortical carcinoma, *BLCA* bladder urothelial carcinoma, *BRCA* breast invasive carcinoma, *CHOL* cholangiocarcinoma, *COAD* colon adenocarcinoma, *DLBC* diffuse large B‑cell lymphoma, *ESCA* oesophageal carcinoma, *GBM* glioblastoma multiforme, *HNSC* head and neck squamous cell carcinoma, *KICH* kidney chromophobe, *KIRC* kidney renal clear cell carcinoma, *KIRP* kidney renal papillary cell carcinoma, *LAML* acute myeloid leukaemia, *LGG* brain lower grade glioma, *LUAD* lung adenocarcinoma, *LUSC* lung squamous cell carcinoma, *MESO* mesothelioma, *OV* ovarian serous cystadenocarcinoma, *PAAD* pancreatic adenocarcinoma, *PCPG* pheochromocytoma and paraganglioma, *PRAD* prostate adenocarcinoma, *READ* rectum adenocarcinoma, *SARC* sarcoma, *SKCM* skin cutaneous melanoma, *STAD* stomach adenocarcinoma, *TGCT* testicular germ cell tumour, *THCA* thyroid carcinoma, *THYM* thymoma, *UCEC* uterine corpus endometrial carcinoma, *UCS* Uterine Carcinosarcoma, *UVM* uveal melanoma.

First, we used a GTEx dataset to analyse the expression levels of physiological BARX2 genes in different normal tissues. As shown in Supplementary Fig. S1A, BARX2 expression was highest in the oesophageal-mucosa, minor salivary glands and vagina and lowest in the left ventricle of the heart, whole blood and tibial nerve. The liver expression levels were in the lower to middle range among those of all normal tissues. Using the TCGA, the mRNA levels of BARX2 were also investigated in 33 tumour tissues (Fig. 1), and all cancers expressed BARX2. The highest BARX2 level was observed in cervical squamous cell carcinoma and endocervical adenocarcinoma (CESC), head and neck squamous cell carcinoma (HNSC) and kidney renal clear cell carcinoma (KIRC). The lowest BARX2 expression was observed in acute myeloid leukaemia (LAML), pheochromocytoma and paraganglioma (PCPG), and uveal melanoma (UVM), and BARX2 expression in LIHC was at a moderate to low. In addition, we analysed the expression of the BARX2 gene in 946 cell lines from 32 tumour types in the CCLE database (Supplementary Fig. S1B). The results showed that the cell lines with the highest expression of the BARX2 gene were breast and colorectal cancer cell lines. Cell lines derived from the haematologic system (e.g. chronic granulocytic leukaemia) showed relatively low BARX2 expression, while lower BARX2 expression was observed in LIHC cell lines.

Subsequently, we compared BARX2 expression levels between tumour and normal samples from 33 cancers in the TCGA and GTEx datasets (Fig. 1). Except for those cancers [ACC, adrenocortical carcinoma; DLBC, diffuse large B-cell lymphoma; LAML, acute myeloid leukaemia; LGG, brain lower grade glioma; MESO, mesothelioma; OV, ovarian serous cystadenocarcinoma; SARC, sarcoma; TGCT, testicular germ cell tumour; THYM, thymoma; UCS Uterine Carcinosarcoma; UVM, uveal melanoma] for which normal tissue data were unavailable or too limited, significant differences in BARX2 expression between tumour and normal tissue were found in 22 types of cancer. Within these types, BARX2 was expressed in bladder urothelial carcinoma (BLCA), cervical squamous cell carcinoma and endocervical adenocarcinoma (CESC), cholangiocarcinoma (CHOL), oesophageal carcinoma (ESCA), kidney renal clear cell carcinoma (KIRC), lung adenocarcinoma (LUAD), and lung squamous cell carcinoma (LUSC) and was upregulated compared to the expression in corresponding normal tissue. In contrast, BARX2 expression was downregulated in breast invasive carcinoma (BRCA), colon adenocarcinoma (COAD), head and neck squamous cell carcinoma (HNSC), rectum adenocarcinoma (READ), and thyroid carcinoma (THCA). However, in kidney chromophore (KICH), kidney renal papillary cell carcinoma (KIRP), pancreatic adenocarcinoma (PAAD), prostate adenocarcinoma (PRAD), stomach adenocarcinoma (STAD), and uterine corpus endometrial carcinoma (UCEC), BARX2 mRNA levels were not significantly different between tumour tissue and corresponding normal tissue.

### Genetic and epigenetic alterations in BARX2 in liver hepatocellular carcinoma

**Figure 2:**
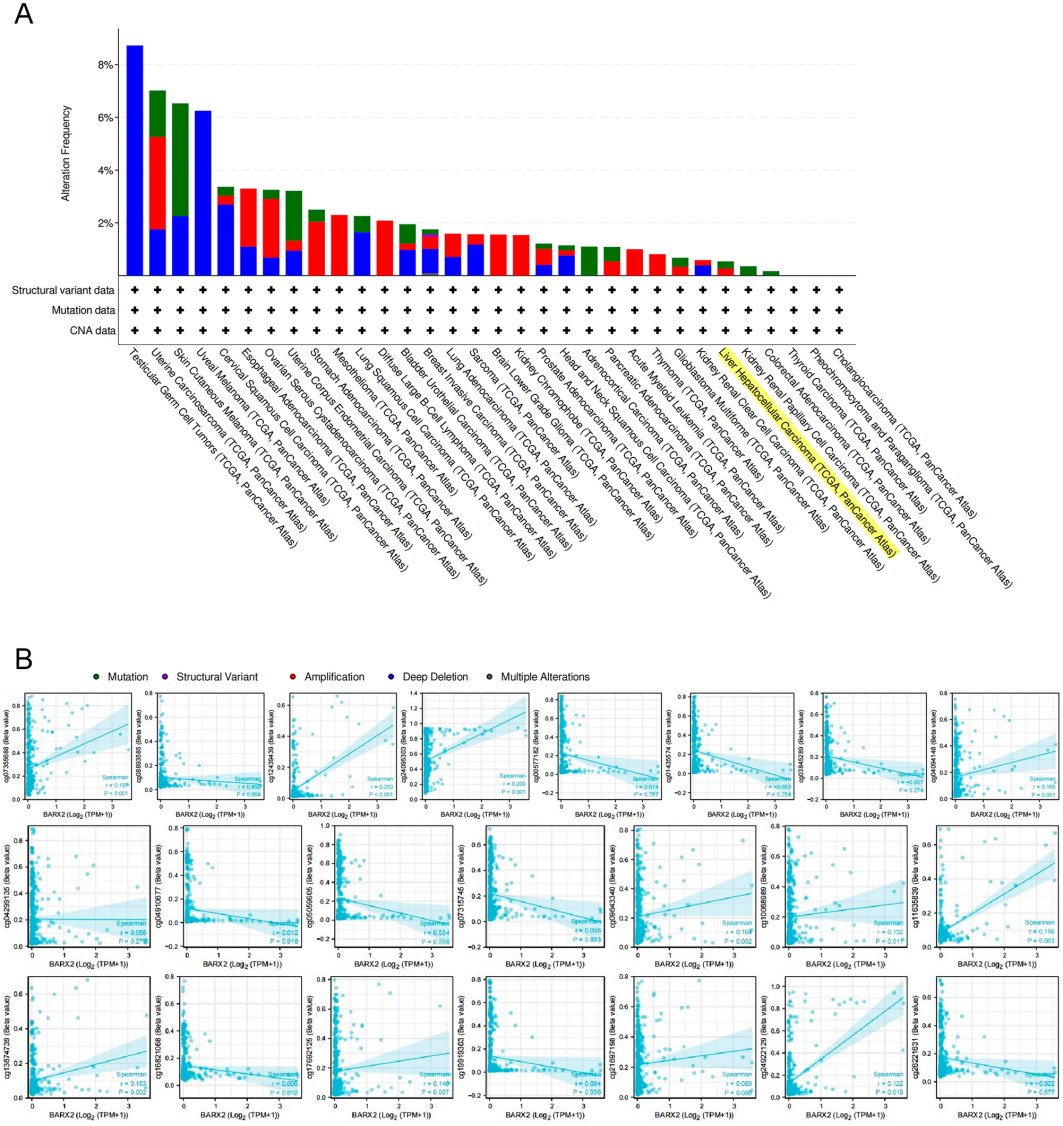
Genetic and epigenetic alterations in BARX2 in LIHC patients from the TCGA dataset. (**A**) cBioPortal analysis of mutations, amplifications and deletions in BARX2 in different tumour tissues. (**B**) SMART App analysis of the correlation between BARX2 methylation levels and gene expression in LIHC patients.

We investigated the genetic alterations in the BARX2 gene in different types of tumours in the TCGA dataset on cBioPortalWeb. As shown in Fig. 2A, the frequency of alterations in the BARX2 gene was highest (8.56%) among TGCT patients with “deep deletion” as the main alteration. “Mutations” and “amplifications” of the BARX2 gene were found to be the main alterations in all cases of hepatocellular carcinoma.

Subsequently, we investigated the BARX2 DNA methylation status of LIHC patients from the TCGA dataset using the SMART App and the MethSurv database. For probes in the promoter region of the BARX2 gene (cg00577182, cg04299135, cg04910677, cg05059605, cg07315745, cg16821068, cg19919303, cg21697198, and cg26221631), the BARX2 DNA methylation level (β value) did not correlate with the expression level of BARX2. However, we observed that hypermethylation of the BARX2 gene body region (cg07355688, cg12439439, cg24095303, cg04094148, cg09643340, cg10068989, cg11635839, cg13874739, cg17692125, cg21697198, and cg24922129) was positively correlated with BARX2 gene expression (Fig. 2B and Supplementary Fig. 2A).

### Multifaceted prognostic value of BARX2 expression in tumour tissues

**Figure 3:**
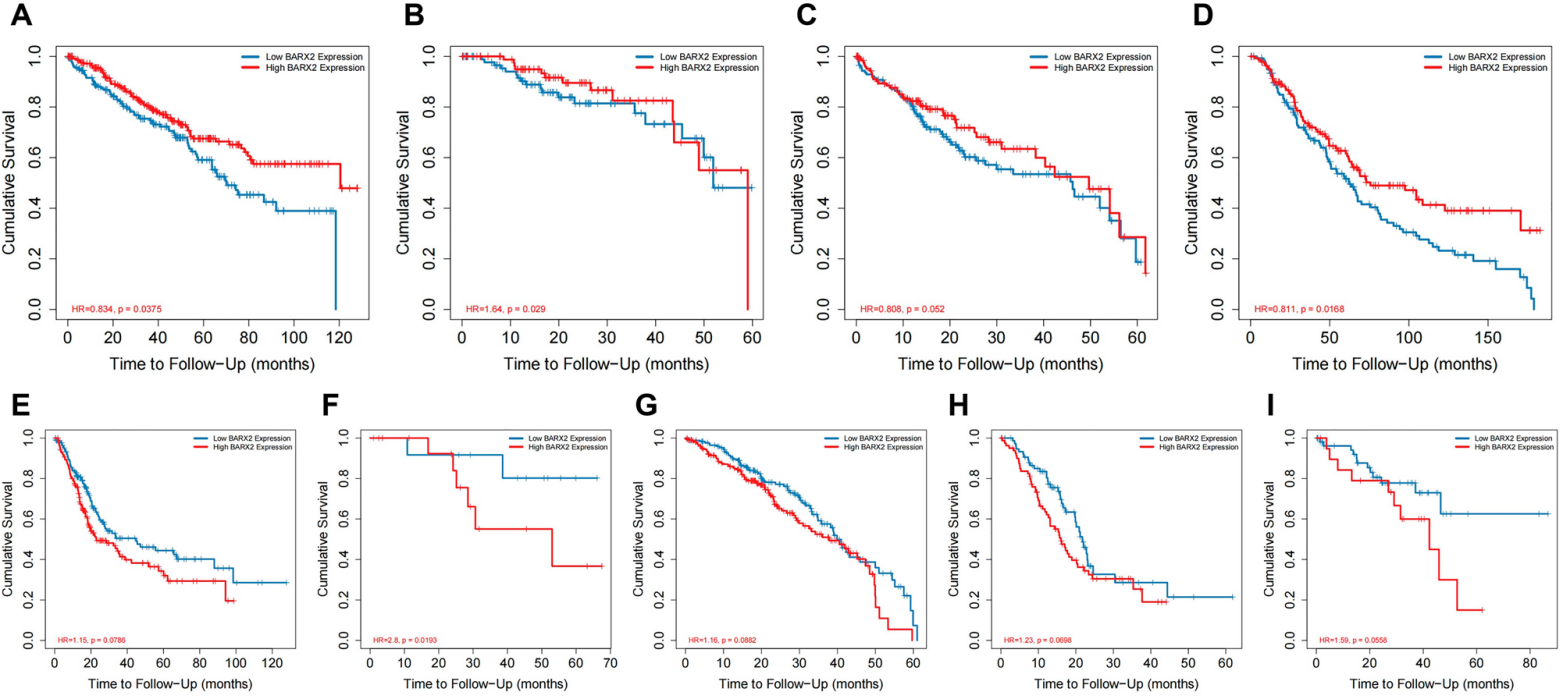
The TIMER 2.0 database was applied to analyse the prognostic value of BARX2 expression for overall survival (OS) in different tumour types. (**A**) KIRC, (**B**) KIRP, (**C**) LIHC, (**D**) SKCM, (**E**) BLCA, (**F**) KICH, (**G**) LUAD, (H) PAAD, (**I**) UVM. *LIHC* liver hepatocellular carcinoma, *BLCA* bladder urothelial carcinoma, *KICH* kidney chromophore, *KIRC* kidney renal clear cell carcinoma, *KIRP* kidney renal papillary cell carcinoma, *LUAD* lung adenocarcinoma, *PAAD* pancreatic adenocarcinoma, *SKCM* skin cutaneous melanoma, *UVM* uveal melanoma.

We investigated the relationship between BARX2 expression and prognosis in cancer patients based on a basic assessment of BARX2 expression in different tumours. By using the TIMER 2.0 database (based primarily on RNA-sequencing data from the TCGA cohort), we found that BARX2 expression was significantly associated with OS in patients with nine cancer types (Fig. 3A–I). In patients with KIRC, KIRP, LIHC or SKCM, upregulation of BARX2 was associated with good OS. In contrast, high expression of BARX2 was associated with poor OS in patients with BLCA, KICH, LUAD, PAAD, or UVM.

### BARX2 is an independent prognostic factor for patients with LIHC

**Figure 4:**
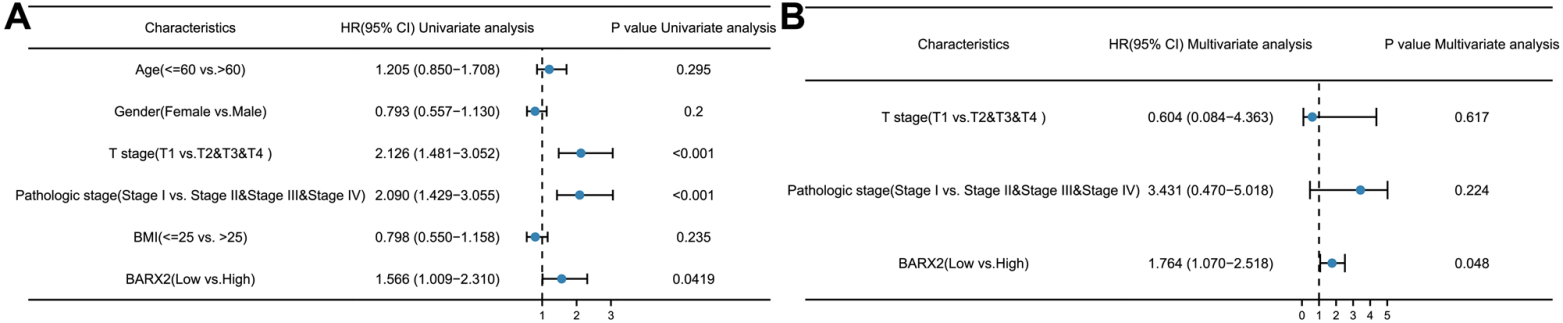
BARX2 overexpression is an independent prognostic factor in patients with LIHC. (**A**) Forest plot for univariate Cox regression analysis of BARX2 mRNA expression with OS in LIHC with different clinicopathological features. (**B**) Forest plot for multivariate Cox regression analysis of BARX2 mRNA expression with OS in LIHC with different clinicopathological features.

Kaplan‒Meier curves and log-rank tests showed that the OS of LIHC patients with high expression of BARX2 in the TIMER 2.0 database was significantly better than that of LIHC patients with low expression, and a Cox proportional risk model was used to confirm the potential of BARX2 as a prognostic factor for LIHC patients from the TCGA dataset. Univariate Cox regression analysis showed that BARX2 expression (low vs. high, HR = 1.566, 95% CI 1.009–2.310, P = 0.0419), T stage (T1 vs. T2&T3&T4, HR = 2.126, 95% CI 1.481–3.052, P = <0.001) and pathologic stage (Stage 1 vs. Stage 2& Stage 3& Stage 4, HR = 2.090, 95% CI 1.429–3.055, P = <0.001) were associated with OS (Fig. 4A). Multivariate Cox regression revealed that BARX2 expression (HR = 1.764, 95% CI 1.070–2.518, P = 0.048) was an independent prognostic factor for OS in LIHC (Fig. 4B).

### BARX2 mRNA expression and clinical features in LIHC

In view of the prognostic significance of BARX2 expression in LIHC and the unclear mechanism of action of BARX2 in hepatocellular carcinogenesis, we focused on investigating the biological role of BARX2 in hepatocellular carcinoma. We summarized the clinical and genetic characteristics of LIHC patients from the TCGA cohort (Table 1). The BARX2low and BARX2High groups of LIHC patients differed in terms of T stage, pathologic stage, residual tumour, cytogenetic risk stratification, and cytogenetic alterations. Low expression of BARX2 was associated with T stage (P = 0.004) and pathologic stage (P = 0.020). In residual tumours, low expression of BARX2 was significantly associated with stage R0, while stage R1 was associated with high expression of BARX2.

**Table 1:** Clinicopathological characteristics of LIHC patients from the TCGA cohort.

Characteristic	Low expression of BARX2	High expression of BARX2	p
n	187	187	
T stage, n (%)			
T1	108 (29.1%)	75 (20.2%)	0.004
T2	36 (9.7%)	59 (15.9%)	
T3	34 (9.2%)	46 (12.4%)	
T4	7 (1.9%)	6 (1.6%)	
Pathologic stage, n (%)			
Stage I	103 (29.4%)	70 (20%)	0.020
Stage II	36 (10.3%)	51 (14.6%)	
Stage III	39 (11.1%)	46 (13.1%)	
Stage IV	2 (0.6%)	3 (0.9%)	
Gender, n (%)			
Female	57 (15.2%)	64 (17.1%)	0.507
Male	130 (34.8%)	123 (32.9%)	
Age, n (%)			
< = 60	89 (23.9%)	88 (23.6%)	1.000
> 60	98 (26.3%)	98 (26.3%)	
OS event, n (%)			
Alive	118 (31.6%)	126 (33.7%)	0.447
Dead	69 (18.4%)	61 (16.3%)	
Race, n (%)			
Asian	83 (22.9%)	77 (21.3%)	0.376
Black or African American	6 (1.7%)	11 (3%)	
White	88 (24.3%)	97 (26.8%)	
Height, n (%)			
< 170	108 (31.7%)	93 (27.3%)	0.408
> = 170	68 (19.9%)	72 (21.1%)	
Histologic grade, n (%)			
G1	33 (8.9%)	22 (6%)	0.294
G2	88 (23.8%)	90 (24.4%)	
G3	56 (15.2%)	68 (18.4%)	
G4	7 (1.9%)	5 (1.4%)	
Residual tumor, n (%)			
R0	172 (49.9%)	155 (44.9%)	< 0.001
R1	2 (0.6%)	15 (4.3%)	
R2	1 (0.3%)	0 (0%)	
Fibrosis ishak score, n (%)			
0	47 (21.9%)	28 (13%)	0.395
1/2	16 (7.4%)	15 (7%)	
3/4	15 (7%)	13 (6%)	
5/6	40 (18.6%)	41 (19.1%)	
Vascular invasion, n (%)			
No	115 (36.2%)	93 (29.2%)	0.121
Yes	50 (15.7%)	60 (18.9%)	
Age, median (IQR)	62 (52, 69.5)	61 (51.25, 68)	0.539

*n* number of patients, *IQR* interquartile range.

**Figure 5:**
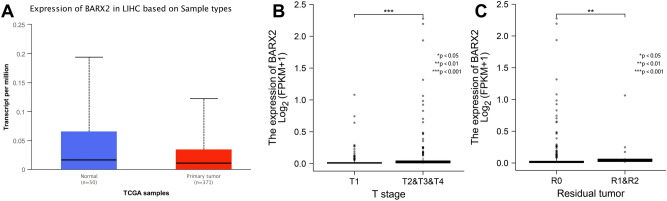
BARX2 expression and clinical features in LIHC patients in the TCGA dataset. (**A**) Comparison of BARX2 expression levels between normal subjects and LIHC patients by UALCAN analysis. (**B**) Effect of T stage on the expression level of BARX2 in LIHC patients. (**C**) Effect of residual tumour on the expression level of BARX2 in LIHC patients.

We also analysed the differential expression of BARX2 in LIHC patients from the TCGA dataset according to T stage and residual tumour status. As shown in Fig. 5A, BARX2 expression differed between normal and LIHC patients, with low expression in LIHC patients and high expression in normal subjects. According to the NCCN staging criteria for LIHC, the expression of BARX2 was lower in patients with LIHC at stage T1 than in those with T2-4 disease (P = 0.001. Fig. 5B). In addition, residual tumour was associated with increased expression of BARX2 (P < 0.001. Fig. 5C).

### BARX2-coexpressed gene analysis in LIHC patients

**Figure 6:**
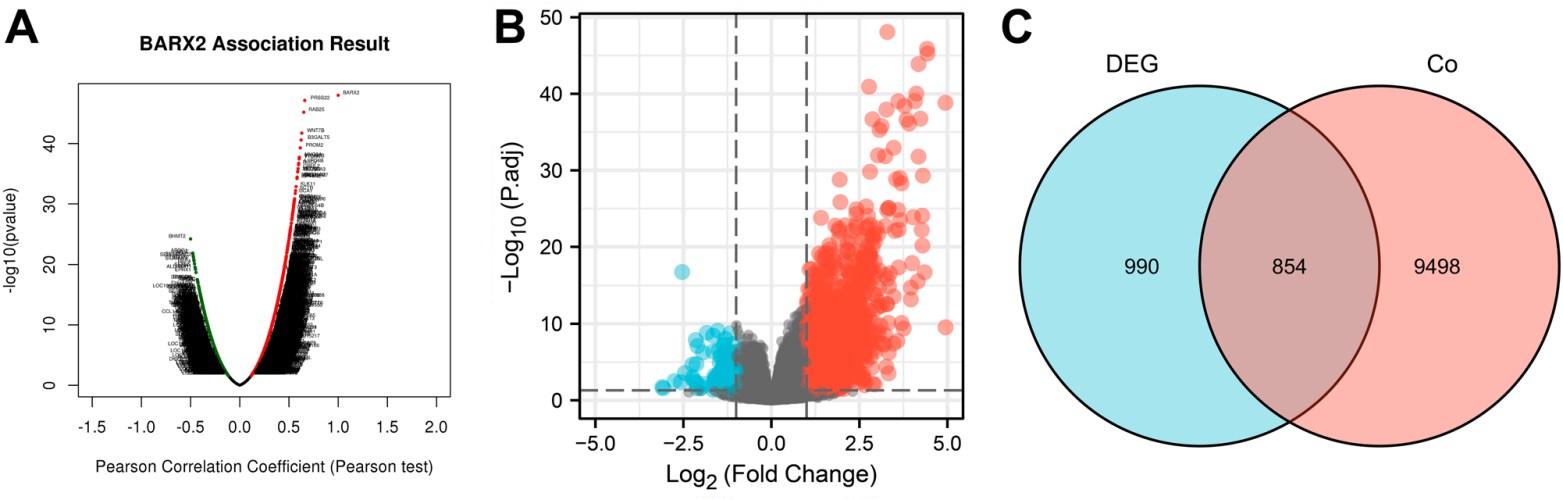
Genome-wide genes associated with BARX2 expression in LIHC. (**A**) Volcano plot analysis of coexpressed genes associated with BARX2 expression using LinkedOmics. (**B**) Volcano plot of differentially expressed genes (DEGs) in the BARX2High and BARX2low groups. (**C**) Venn diagram of overlapping genes between the BARX2 significantly coexpressed genes and significant DEGs.

To further investigate the mechanism underlying the role of BARX2 in the development of LIHC, the genes coexpressed with BARX2 in LIHC patients in the TCGA database were first investigated using LinkedOmics (Fig. 6A). The results indicated that a total of 10352 coexpressed genes were significantly correlated with BARX2 in LIHC (FDR < 0.05, P < 0.05, and |cor.| ≥ 0.3, Supplementary Table S1). Among these 10,352 genes, 4899 were positively correlated with BARX2 expression, and 5453 were negatively correlated with BARX2 expression. Subsequently, DEGs were also identified between the BARX2high and BARX2low groups in LIHC. As shown in Fig. 6b and Supplementary Table S2, a total of 1844 DEGs were identified (P<0.05, |log2Fc|≥1). These 1844 BARX2-associated DEGs were compared with the 10,352 significantly coexpressed genes mentioned above by the online ventrogram tool, and 854 overlapping genes were identified (Fig. 6C, Supplementary Table S3) for further functional analysis.

### Functional enrichment analysis of BARX2 in LIHC

**Figure 7:**
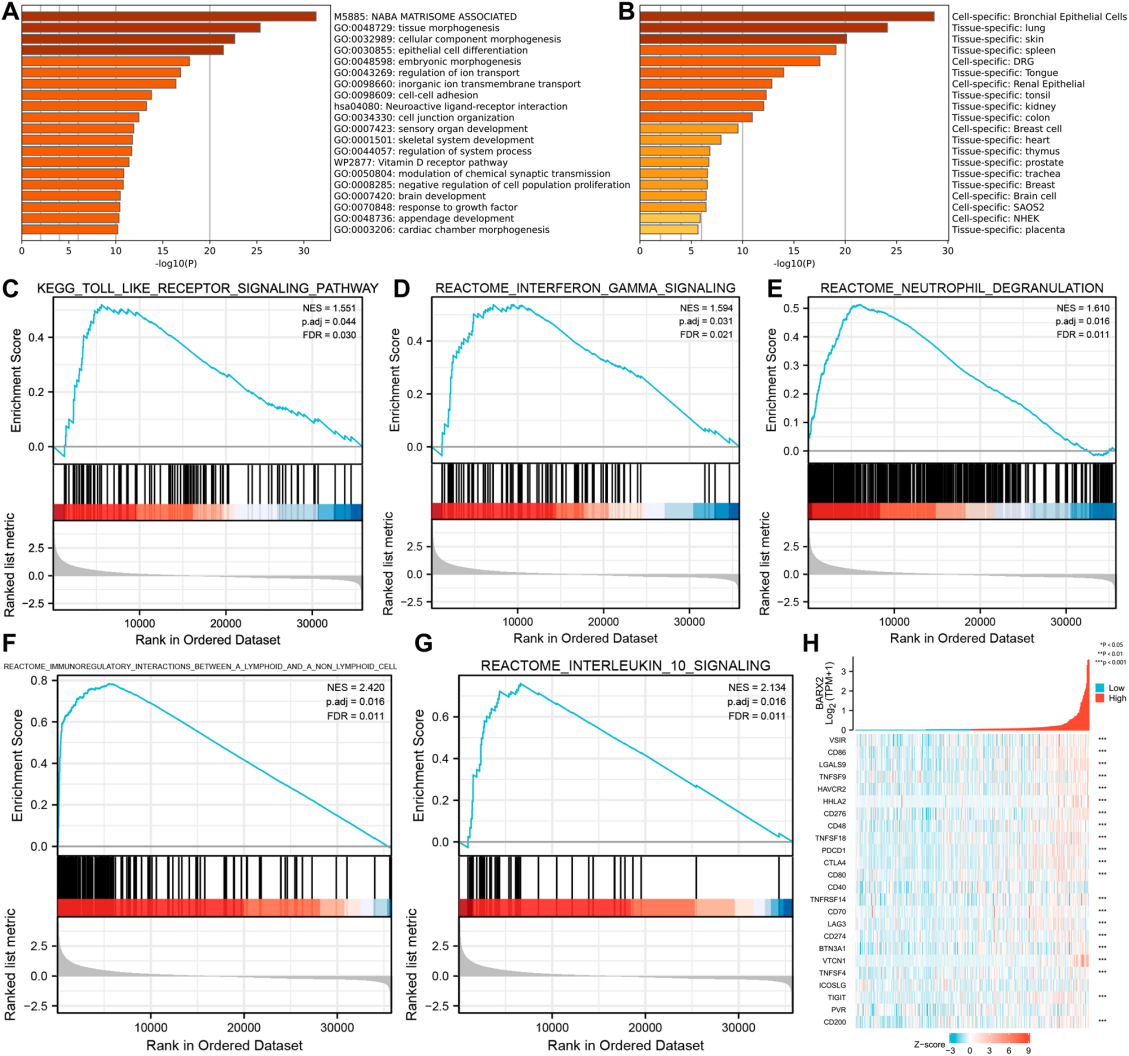
Functional enrichment analysis of overlapping genes in LIHC. (**A**) Enrichment analysis of GO terms and KEGG pathways associated with BARX2 expression analysed by Metascape. (**B**) Enrichment analysis of overlapping genes in tissues and cells analysed by Metascape. (**C–G**) Signalling pathways enriched in GSEA of overlapping genes. (**H**) Coexpression of BARX2 and immune‑related genes in LIHC.

To investigate the biological functions of the 854 overlapping genes, GO/KEGG analyses were performed using the Metascape database. The top 20 genes with the highest enrichment are shown in Fig. 7A. Concentration analysis results indicated that BARX2 and its related partners were functional mediators of growth and development, including naba matrisome associated, tissue morphogenesis, cellular component morphogenesis, epithelial cell differentiation, embryonic morphogenesis, cell‒cell adhesion, sensory organ development, and skeletal system development. These genes were also associated with immunomodulatory transport regulation, including regulation of ion transport, inorganic ion transmembrane transport, regulation of system process, modulation of chemical synaptic transmission, and negative regulation of cell population proliferation. In addition, BARX2 expression was associated with the response to growth factors and neuroactive ligand‒receptor interactions. On the other hand, as shown in Fig. 7B, the overlapping genes were abundant in the thymus, spleen and lungs, providing further evidence for an immunomodulatory role for BARX2 in the pathogenesis of LIHC. We performed GSEA by using GSEA software to further explore the molecular pathways that were significantly altered between the BARX2high and BARX2low groups in LIHC. The data suggested that BARX2 primarily positively regulated immune-related processes or pathways, including the Toll-like receptor signalling pathway (Fig. 7C), interferon gamma signalling (Fig. 7D), neutrophil degranulation (Fig. 7E), immunoregulatory interactions between a lymphoid and a non-lymphoid cell (Fig. 7F), and interleukin-10 signalling (Fig. 7G), further suggesting an immunological role for BARX2 in LIHC occurrence.

Enrichment analysis showed that BARX2 was involved in the immune network of LIHC; therefore, the correlations between BARX2 expression in LIHC patients and immune-related genes were explored by gene coexpression analysis. The analysed genes encoded immune checkpoint molecules. As shown in Fig. 7H, BARX2 expression was associated with the expression of most immune checkpoint-related genes, including VSIR, CD48, LAG3, and CD200.

### PPI networks of BARX2-related partners

**Figure 8:**
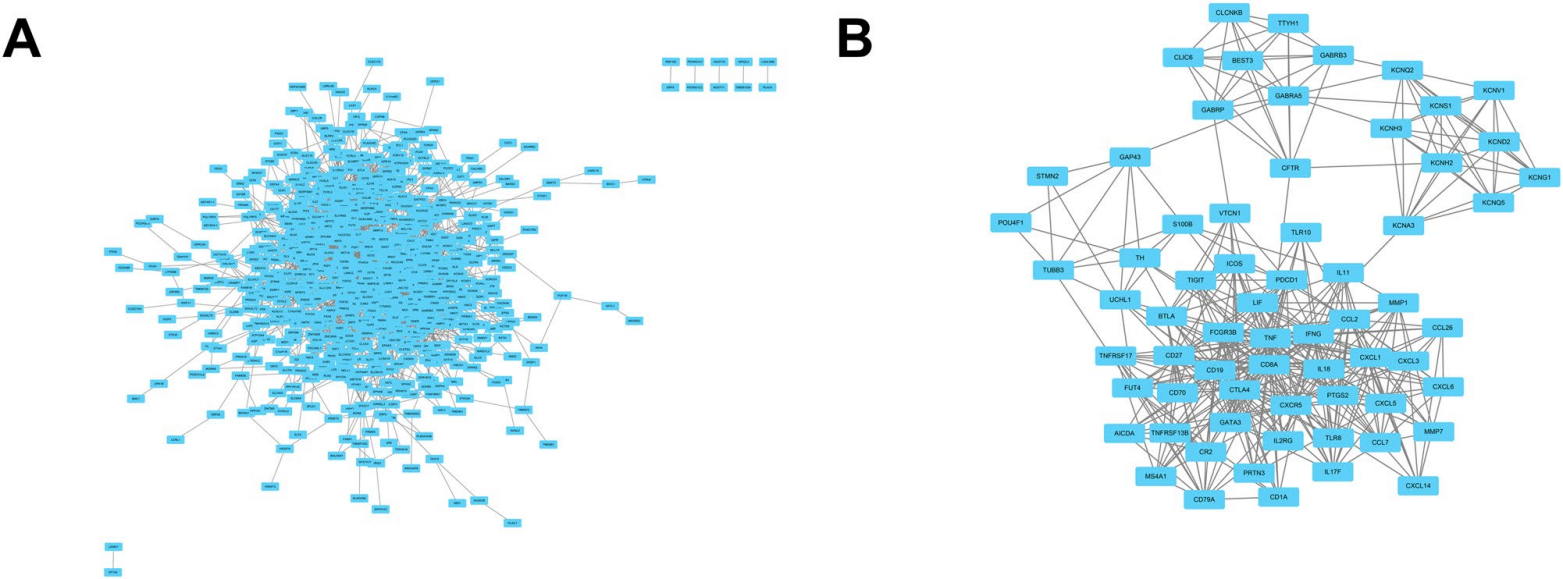
Protein‒protein interaction (PPI) network of overlapping genes in LIHC. (**A**) PPI network for overlapping genes, analysed by STRING. (**B**) Network of hub genes screened from overlapping genes by MCODE analysis with Cytoscape software.

The PPI network of the overlapping genes was analysed with the STRING database. The PPI network diagram generated by Cytoscape software is shown in Fig. 8A. When the 854 overlapping genes plus BARX2 were used as the input, a total of 760 nodes and 3147 edges were obtained. Subsequently, the PPI network was further analysed using the MCODE plugin in Cytoscape software to screen for hub genes. MCODE analysis showed that the most significant module (MCODE Score=13.015) consisted of 66 hub genes (Fig. 8B), all of which were upregulated in LIHC. Among these hub genes, 14 genes including CCL26, CD1A, CLCNKB, CXCL1, CXCL3, CXCL5, IL2RG, IL18, KCND2, KCNG1, MMP1, MMP7, STMN2 and TUBB3 were significantly associated with inferior OS in AML patients (Supplementary Fig. S4).

### The roles of BARX2 in the regulation of immune cell infiltration

**Figure 9:**
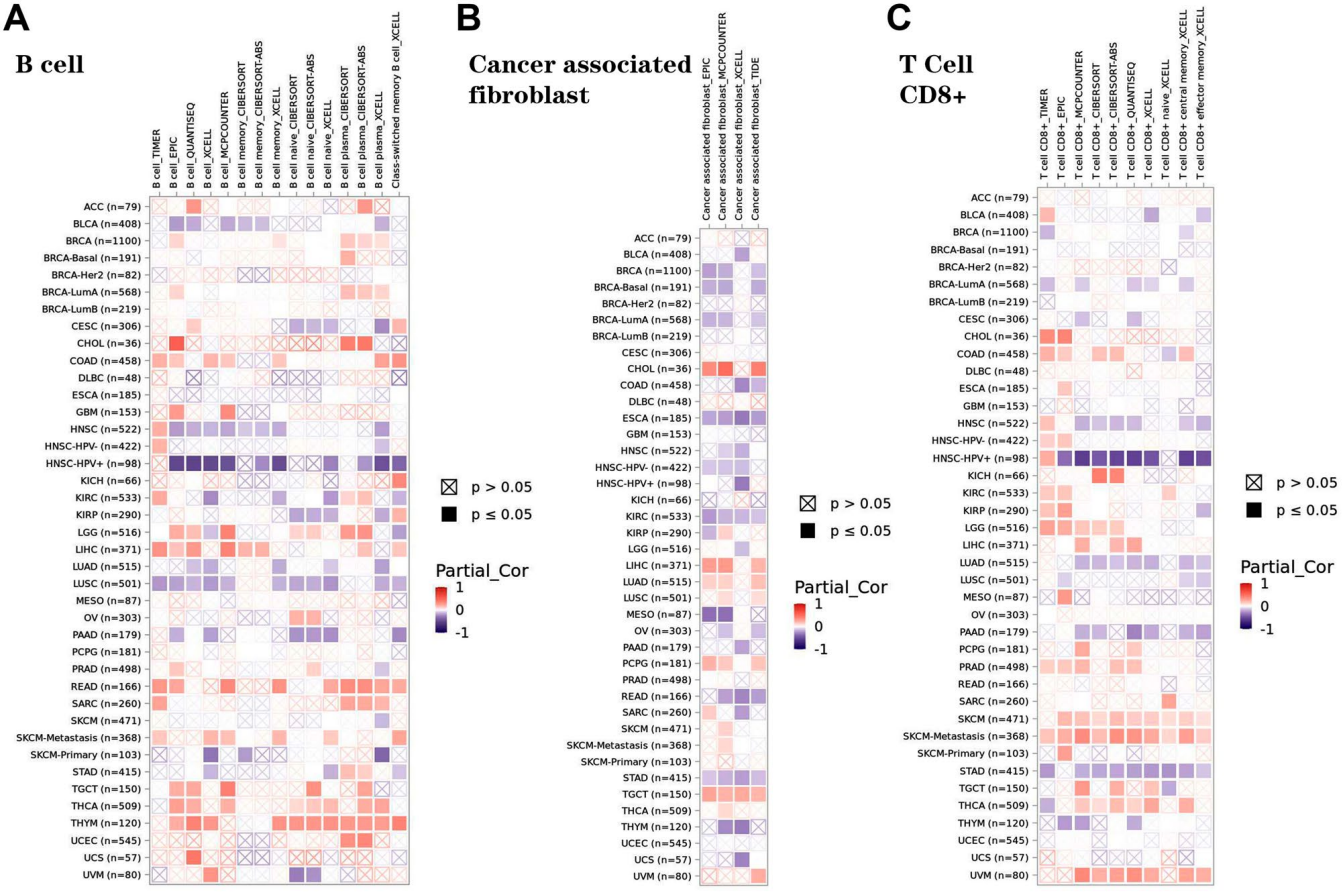
Correlations of immune cells with BARX2 expression in tumours. (**A–C**) Characterization of BARX2 expression in relation to (**A**) B-cell, (**B**) cancer-associated fibroblast and (**C**) CD8 + T-cell immune infiltration using the TIMER2.0 database. Several algorithms, such as TIMER, EPIC, QUANTISEQ, XCELL, MCPCOUNTER, CIBERSROT, CIBERSORT-ABS and TID, were used to explore these correlations. Red indicates positive correlations (0–1), blue indicates negative correlations (− 1 to 0). *p < 0.05 is considered statistically significant. Crosses indicate nonsignificant correlations.

In recent years, relevant scientific studies have found that immune infiltration is associated with the occurrence, development and metastasis of human cancers. The TIMER, EPIC, QUANTISEQ, XCELL, MCPCOUNTER, CIBERSORT, CIBERSORT-ABS and TIDE algorithms were applied to explore the correlations between BARX2 expression and invasion of different immune cells in tumour tissues from multiple cancers. B-cell infiltration in COAD, LIHC, READ and THYM was positively correlated with BARX2 expression (Fig. 9A). Furthermore, in ESCA, KIRC and STAD, infiltration of cancer-associated fibroblasts was negatively correlated with the expression of BARX2 (Fig. 9B). The expression of BARX2 in SKCM was positively correlated with the infiltration of CD8+ T cells (Fig. 9C). We found no significant correlations between BARX2 expression and the infiltration values of endothelial cells, eosinophils, macrophages, monocytes, or natural killer cells (NK) (Supplementary Fig. S4). These findings suggest that BARX2 may act as a novel biomarker of immune-related tumorigenesis.

### Expression pattern of BARX2 at the single cell level

**Figure 10:**
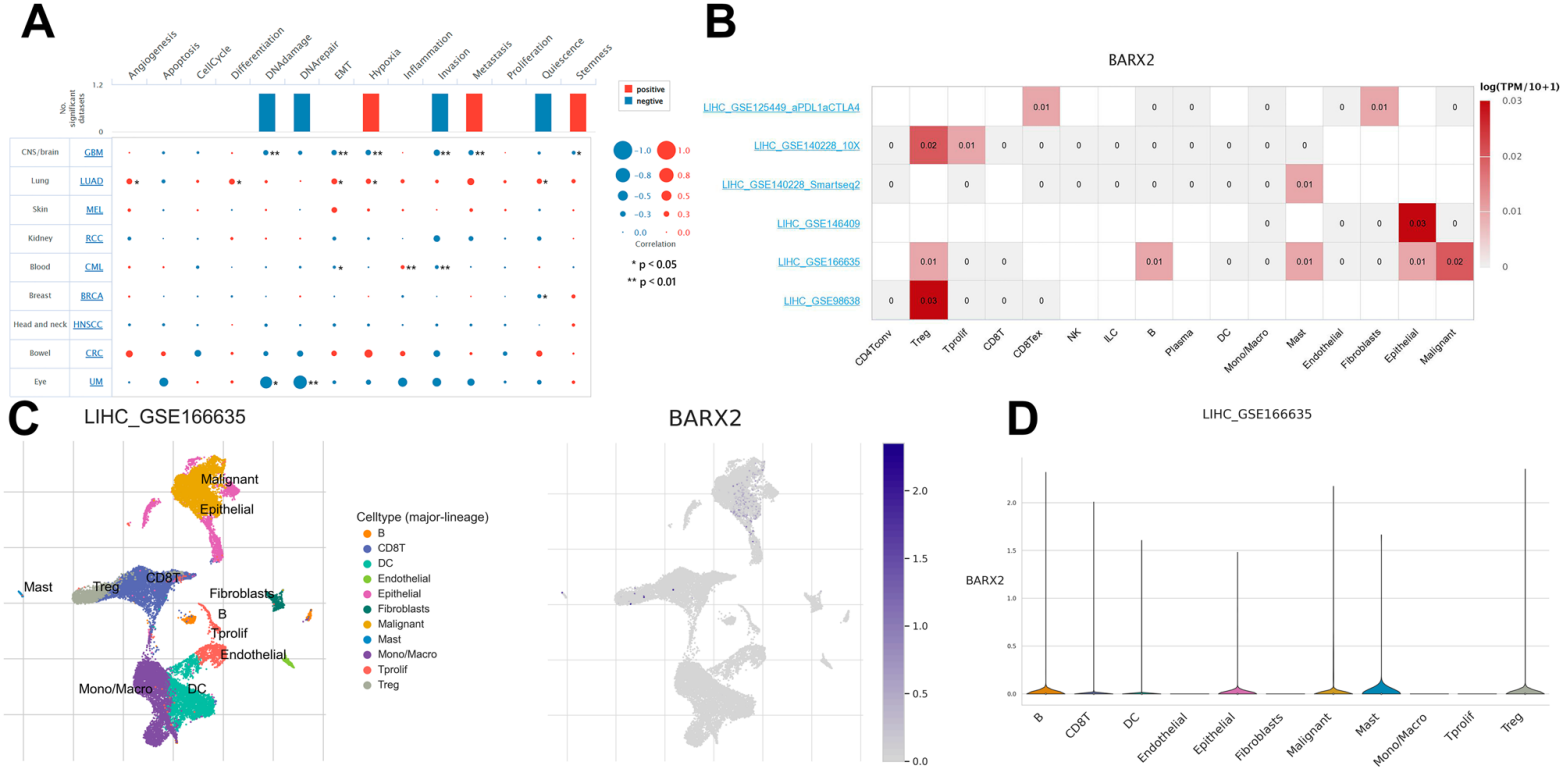
(**A**) The relationships between BARX2 expression and different functional states in tumours were explored by the CancerSEA tool. *p < 0.05, **p < 0.01. (**B**) BARX2 expression in different cell types from different hepatocyte datasets. (**C,D**) Single-cell analysis of BARX2 in the LIHC_GSE166635 dataset.

The key technology single-cell transcriptome sequencing offers great potential for analysing the potential function of candidate molecules at the single-cell level. In chronic myeloid leukaemia (CML), BARX2 expression was negatively correlated with EMT and invasion. In contrast, BARX2 expression was positively correlated with inflammation. BARX2 expression in glioblastoma multiforme (GBM) and uveal melanoma (UM) was negatively associated with almost all tumour biological behaviours, such as DNA repair, DNA damage, invasion and metastasis. BARX2 expression in lung adenocarcinoma (LUAD) was positively associated with almost all tumour biological behaviours, such as angiogenesis, differentiation, EMT and hypoxia (Fig. 10A). We used the TISCH2 database to analyse BARX2 expression in different cell types from different hepatocellular carcinoma datasets. The results showed that BARX2 expression was higher in regulatory T cells (Tregs), mast cells (Mast) and epithelial cells in various hepatocellular carcinoma datasets (Fig. 10B). For single-cell analysis, we selected the LIHC_GSE166635 dataset and employed the TISCH2 tool to understand the comparative expression of BARX2 in different cell populations. The results showed that BARX2 was mainly expressed in B cells, CD8+ T cells and mast cells (Fig. 10C,D) (***P<0.001, **P<0.01, *P<0.05).

## Discussion

Currently, the clinical diagnosis and treatment of hepatocellular carcinoma are well established; hepatocellular carcinoma, the main type of liver cancer, has a poor prognosis with a five-year survival rate of no more than 20% due to its potential for recurrence and metastasis. Therefore, there is an urgent need to explore new biomarkers to improve the prognosis of patients with hepatocellular carcinoma. Numerous studies have shown that BARX2 plays important regulatory roles in processes such as inflammation, angiogenesis, cell proliferation and differentiation^13–15,22,25–27^. Dysregulation of BARX2 is associated with infectious diseases and cancer^24,28–31^. However, there are few studies on the role of BARX2 in the occurrence of LIHC^28^. Therefore, in this study, the expression and prognostic value of BARX2 in multiple cancers was comprehensively explored using publicly available datasets. In addition, the clinical significance and role of BARX2 in LIHC were explored for the first time by bioinformatic analysis and functional analysis. There were some observations in this study.

First, the role of BARX2 in tumorigenesis may be different among different cancers. Although BARX2 expression is upregulated in various types of tumours, such as lung adenocarcinoma, ovarian serous adenocarcinoma and nasopharyngeal cancers^25,32,33^, the current study shows that BARX2 is also expressed downregulated in different types of tumours such as breast, gastric and colon cancers^24,29,31^, suggesting that BARX2 expression is tissue specific. However, Stevens and Meech et al. found increased expression of BARX2 in breast cancer tissues^31^, with upregulation of the expression of MMP9 and inhibitor of metalloproteinases 4 (TIMP4), which ultimately promoted invasion and migration of breast cancer cells, contradicting our current results, probably due to differences in the tumour tissue subtypes studied. On the other hand, the prognostic value of BARX2 expression in cancer patients has been rarely reported thus far^34^. In addition, a PPI network of BARX2-related partners was constructed, and 66 hub genes were ultimately screened. Among these hub genes, 14 genes had an impact on the OS of LIHC patients (Supplementary Fig. S3).

In this study, the expression and prognostic value of BARX2 in multiple cancers were comprehensively explored using publicly available datasets. The present study, based on the TCGA and GEO databases, shows the multifaceted prognostic impact of BARX2 overexpression on OS in certain tumour types, with higher BARX2 expression associated with better OS in KIRC, KIRP, LIHC, and SKCM and poorer OS in BLCA, KICH, LUAD, PAAD, and UVM. Taken together, these data suggest that BARX2 expression levels and prognostic significance are highly cancer dependent, requiring further confirmation of the specific role of BARX2 in each cancer. In addition, the clinical significance and role of BARX2 in LIHC were explored for the first time by bioinformatic analysis and functional analysis. LIHC patients with BARX2 overexpression exhibited longer OS in the TCGA dataset. Analysis of clinical parameters in LIHC patients from the TCGA cohort also suggested that upregulation of BARX2 was an independent novel prognostic biomarker for OS in LIHC. We further explored the relationships between BARX2 expression and clinical or genetic phenotypes of patients with LIHC. The results showed that the expression of BARX2 was reduced in LIHC patients, whereas it was higher in normal subjects. Importantly, BARX2 overexpression was associated with T stage and residual tumour. Overall, these data imply a critical role for BARX2 in the pathogenesis of LIHC.

In addition, we analysed the genetic and epigenetic alterations in BARX2 in LIHC. “Mutations” and “amplifications” of the BARX2 gene were found to be the main alterations in all cases of LIHC. For DNA methylation, we observed a significant association between increased DNA methylation levels in the genomic region and high expression of BARX2. However, the exact mechanism has not been elucidated. Numerous studies have shown that DNA methylation has an impact on prognosis in different cancers^35,36^. The association of high BARX2 expression with poor OS may be due to gene body hypermethylation, and hypermethylation of BARX2 in the gene body region is correlated with poor OS in LIHC patients. Therefore, in addition to BARX2 expression, BARX2 methylation on the gene body can be considered a potential prognostic biomarker for LIHC.

It has previously been reported that BARX2 is abundant in immune cells such as lymphocytes, endothelial cells, and glandular cells and is involved in the immune response during early tissue development. This study shows for the first time by GO/KEGG and GSEA analyses that the role of BARX2 in LIHC development is mainly related to the positive regulation of immune responses, such as the Toll-like receptor signalling pathway, interferon gamma signalling, neutrophil degranulation, immunomodulatory interactions between lymphocytes and nonlymphoid cells, and interleukin-10 signalling. We further explored the molecular pathways that were significantly altered between the BARX2-high and BARX2-low groups in the LIHC dataset by GSEA. The results showed that BARX2 mainly positively regulated immune-related processes or pathways, including the Toll-like receptor signalling pathway (Fig. 7C), interferon gamma signalling pathway (Fig. 7D), neutrophil degranulation (Fig. 7E), immunomodulatory interactions between lymphocytes and nonlymphoid cells (Fig. 7F), and interleukin-10 signalling pathway (Fig. 7G). Enrichment analysis showed that BARX2 was involved in the immune network of LIHC. BARX2 expression correlated with the expression of most immune checkpoint-related genes, including VSIR, CD48, LAG3 and CD200. Overall, BARX2 has an immunological role in LIHC development.

It has also been demonstrated that BARX2 expression in a variety of tumour cells is negatively correlated with the biological behaviour of tumours, such as glioblastoma multiforme (GBM) and uveal melanoma; however, expression in lung adenocarcinoma (LUAD) was positively correlated with tumour biological behaviour (Fig. 10A). Our comprehensive bioinformatic analysis of BARX2 expression in different cell types from different liver cancer datasets showed that BARX2 expression was higher in regulatory T cells (Tregs), mast cells (Mast) and epithelial cells (Fig. 10B–D).

In this study, the clinical significance and role of BARX2 in LIHC were first investigated by bioinformatic analysis, and the role and mechanism of BARX2 in LIHC occurrence were predicted by enrichment analysis^14,26,37,38^. However, its specific mechanism of action and clinical application need to be further validated.

Methods

### Downloading and processing of data from public databases

We downloaded The Cancer Genome Atlas (TCGA) gene expression RNA-seq data for 31 normal tissues (containing 10363 TCGA tumour tissues and 730 TCGA normal tissues representing 33 types of cancer) and the Genotype-Tissue Expression (GTEx) gene expression RNA-seq data using UCSC Xena (https://xena.ucsc.edu/). We used Toil software to process the raw RNA-seq data and extract BARX2 gene expression data from the GTEx and TCGA datasets for subsequent analysis^39^. The gene expression profiles of the GSE36376 dataset in the Gene Expression Omnibus (GEO) database (http://www.ncbi.nlm.nih.gov/geo/), containing RNA-seq data for 433 LIHC patients^40^, and the Beat LIHC dataset (http://www.vizome.org) were also used to investigate the relationships between BARX2 mRNA expression and clinicopathological factors in LIHC patients. Expression analysis of BARX2 in tumour and normal tissues was performed with The UALCAN database (http://ualcan.path.uab.edu/); the dataset contained RNA-seq and clinical data for 33 cancer types in the TCGA dataset^41^. We used it to analyse the expression of BARX2 in different types of tumour samples and to download box plots from the UALCAN website. The GTEx portal contained RNA-seq data for 54 nonlesional tissue loci from nearly 1000 individuals^42^. We used it to analyse the expression of BARX2 in different normal tissues and download box plots from the GTEx portal. To study the difference in BARX2 expression between tumour and normal tissues from the GTEx and TCGA datasets, if each group of data was normally distributed, a two-sample Student’s t test was used; otherwise, the Mann‒Whitney U test was used. RNA-seq data were normalized by log2 transformation. We normalized RNA-seq data by log2 transformation and analysed the data with IBM SPSS Statistics 28 software.

### BARX2 analysis of available cell lines in the Cancer Cell Line Encyclopedia (CCLE) database

We obtained RNA-seq data, DNA methylation data, gene mutation data and copy number data for human cancer cell lines from the CCLE database (https://portals.broadinstitute.org/ccle)^43^, compared BARX2 expression levels among different cancer cell lines and downloaded box plots from the CCLE website.

### Survival analysis

TIMER 2.0 (http://timer.comp-genomics.org)^44^ is commonly used to explore the prognostic significance of genes in different types of cancer. We used this database to explore the prognostic value of BARX2 expression on overall pancancer survival. Kaplan‒Meier survival analysis and the log-rank test were used to calculate p values^45^.

### Genetic variation analysis

We used the cBioPortal website (https://www.cbioportal.org) for pancancer analysis of BARX2 gene alterations according to the online instructions^46,47^. The results of genetic alteration characteristics of the BARX2 gene, such as frequency, mutation type and copy number alteration (CNA), among different tumours in the TCGA database are displayed in the “Cancer Types Summary” module of the cBioPortal website.

### BARX2 DNA methylation analysis

We analysed BARX2 DNA methylation levels in LIHC patients through two public databases, the Shiny Methylation Analysis Resource Tool (SMART) App database (http://www.bioinfo-zs.com/smartapp/)^48^ and the MethSurv database (https://biit.cs.ut.ee/methsurv/)^49^, which contains Infinium Human Methylation 450 K BeadChip data, RNA-seq data, and clinical data for 33 cancer types from the TCGA dataset. The relationships between BARX2 DNA methylation levels or gene expression and the prognostic value of OS in LIHC patients were then investigated.

### Analysis of coexpressed genes and differentially expressed genes (DEGs)

We used the LinkedOmics database (http://www.linkedomics.org/login.php)^50,51^ to detect coexpressed genes associated with BARX2 expression in RNA-seq data for LIHC patients in the TCGA cohort. We calculated Pearson correlation coefficients and generated volcano plots for the coexpressed genes on the LinkedOmics website. The heatmap we generated via the Limma package in R 4.2.2 shows results for the coexpression of BARX2 and immune-related genes.

We divided the LIHC patients from the TCGA dataset into two groups (BARX2 low and BARX2 high) based on the median level of the BARX2 gene from the RNA-seq data. We used the Limma package in R4.2.2 to screen and plot DEGs in volcano plots comparing the BARX2-low and BARX2-high groups of LIHC patients. Then, we used the Draw Venn diagrams online tool (http://bioinformatics.psb.ugent.be/webtools/Venn/)^52^ to explore the overlapping genes between the DEGs and coexpressed genes, which laid the foundation for further analyses.

### Functional enrichment analysis

We used the Metascape database (http://metascape.org/gp/index.html#/main/step1) to perform functional enrichment analysis of the screened overlapping genes, including Gene Ontology (GO) and Kyoto Encyclopedia of Genes and Genomes (KEGG) pathway and tissue enrichment analyses^53–56^.

### Gene set enrichment analysis (GSEA)

We used the GSEA v4.1.0 database (www.broadinstitute.org/gsea)^57^ to perform gene set enrichment analysis (GSEA) of the screened overlapping genes and identified abundant signalling pathways associated with LIHC^58–60^. For all GSEA analyses, “c2.cp.kegg.v7.1.symbs.gmt” was selected from the MSigDB genome as the reference genome. We performed statistical analysis of the enriched signalling pathways with normalized P<0.05, false discovery rate (FDR) q<0.25, and normalized enrichment score (NES) >1.5 used to define statistically significant enrichment.

### Protein‒protein interaction (PPI) Analysis

We used the STRING database (https://string-db.org/) for PPI network analysis to build a PPI network based on the DEGs in the circRNA–miRNA‒mRNA network^61^. Visualization of the network was performed using Cytoscape. Then, the plugin MCODE in Cytoscape_v3.9.1 software was used for visualization of PPI networks and identification of hub genes in the PPI networks^62–64^. “MCODE” is a Cytoscape plugin that has been used to identify important modules and hub genes with the cut-off criteria of degree ≥ 2 and k-core ≥ 2.

### Infiltration of immune cells

We applied the TIMER 2.0 tool (http://timer.cistrome.org/), which includes multiple algorithms such as TIMER, EPIC, MCPCOUNTER, XCELL and TIDE, to assess the correlations of BARX2 expression with the levels of immune infiltration in different TCGA cancers.

### Single-cell sequencing

cBioPortal (https://www.cbioportal.org/)^64^ was used to collect information on the frequency of alterations, mutation types, mutation sites and three-dimensional (3D) structures of candidate proteins in all TCGA tumours. For single-cell datasets, the TISCH2 tool (http://tisch.comp-genomics.org/home/) provides meta-information, cell type annotations, expression visualization and differential gene expression information.

### Statistical analysis

We used the χ^2^ test to evaluate the correlations between BARX2 expression and clinicopathological parameters such as the French-American-British (FAB) classification, sex, cytogenetic risk, and chromosomal alterations. A t test or ANOVA was performed to evaluate intergroups differences in continuous variables with a normal distribution such as age, BARX2 gene expression level, cell cycle assay results, and Annexin V-APC and PI staining results. Otherwise, the Mann‒Whitney U test or Kruskal‒Wallis test was used. All P values were bilateral, and P < 0.05 was considered statistically significant. We used IBM SPSS 28.0 to perform statistical analysis (***P < 0.001, **P < 0.01, *P < 0.05).

## Supplementary Information

Supplementary Legends.

Supplementary Figure S1.

Supplementary Figure S2.

Supplementary Figure S2.

Supplementary Figure S4.

Supplementary Tables.

## Data Availability

The datasets obtained from web-based sources and subsequently analysed in our study were from The Cancer Genome Atlas (TCGA) database, UCSC Xena (https://xena.ucsc.edu/), CCLE database (https://portals.broadinstitute.org/ccle), TIMER 2.0 database (http://timer.compgenomics.org), cBioPortal web database (https://www.cbiop ortal.org/), Shiny Methylation Analysis Resource Tool (SMART) App (http://www.bioinozs.com/ smartapp/) database, and MethSurv database (https://biit.cs.ut.ee/methsurv/). The gene expression profile of the GSE14468 dataset from the Gene Expression Omnibus (GEO) database (http://www.ncbi.nlm.nih.gov/geo/) was also evaluated.
