# Supplementary material for: Pancancer analysis of oncogenic BARX2 identifying its prognostic value and immunological function in liver hepatocellular carcinoma
Source: Sci Rep. 2023 May 9;13:7560. doi: 10.1038/s41598-023-34519-8 (PMC10170086; doi:10.1038/s41598-023-34519-8)
Supplement: Supplementary file 1 — Supplementary Legends. [file 41598_2023_34519_MOESM1_ESM.docx]

Pancancer analysis of oncogenic BARX2 identifying its prognostic value and immunological function in liver hepatocellular carcinoma

Shi’an Yu1,+,Yu Yang2,+ ,Han’qing Yang1,+ ,Long Peng2 ,Zhi’peng Wu1,Liang Sun1 Zheng’yi Wu1 ,Xu’zhe Yu1, and Xiang’bao Yin1,*

**Additional file 1: Supplementary Figure S1.** Expression of BARX2 in normal tissues and cancer cell lines. (A) BARX2 expression in normal tissues, analyzed by GTEx portal; (B) BARX2 expression in cancer cell lines analyzed by CCLE.

**Additional file 2: Supplementary Figure S2.** Epigenetic alterations of BARX2 in LIHC. The exact location of the CpG locus in the BARX2 gene body was obtained from the UCSC database.

**Additional file 3: Supplementary Figure S3.** Prognostic value of hub genes for OS in LIHC patients.

**Additional file 4: Supplementary Figure S4.** The relationship between BARX2 expression and immune infiltration of (A) Endothelial cell, (B) Eosinophil, (C) macrophage, (D) monocyte, (E)NK was depicted by TIMER2.0 database.

**Supplementary Table S1.** The co‑expressed genes associated with BARX2 expression in LIHC.

**Supplementary Table S2.** The differentially expressed genes between the BARX2high and BARX2low group of LIHC patients.

**Supplementary Table S3.** The overlapping genes between significantly co‑expressed genes and significantly differentially expressed genes of BARX2 in LIHC.
