# Supplementary figures and images for: Pancancer analysis of oncogenic BARX2 identifying its prognostic value and immunological function in liver hepatocellular carcinoma
Source: Sci Rep. 2023 May 9;13:7560. doi: 10.1038/s41598-023-34519-8 (PMC10170086; doi:10.1038/s41598-023-34519-8)

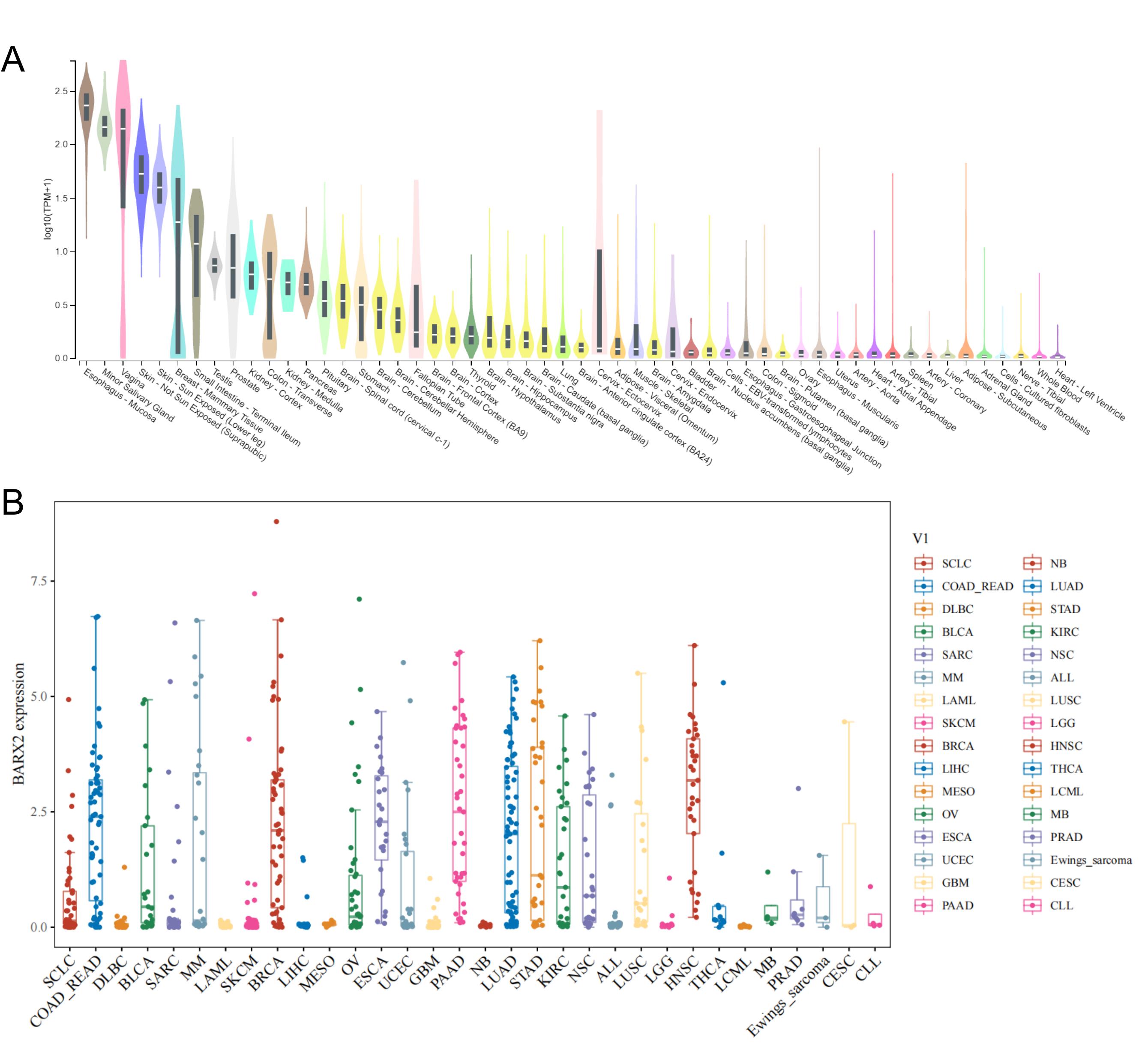

Supplement: Supplementary file 2 — Supplementary Figure S1. [file 41598_2023_34519_MOESM2_ESM.jpg]

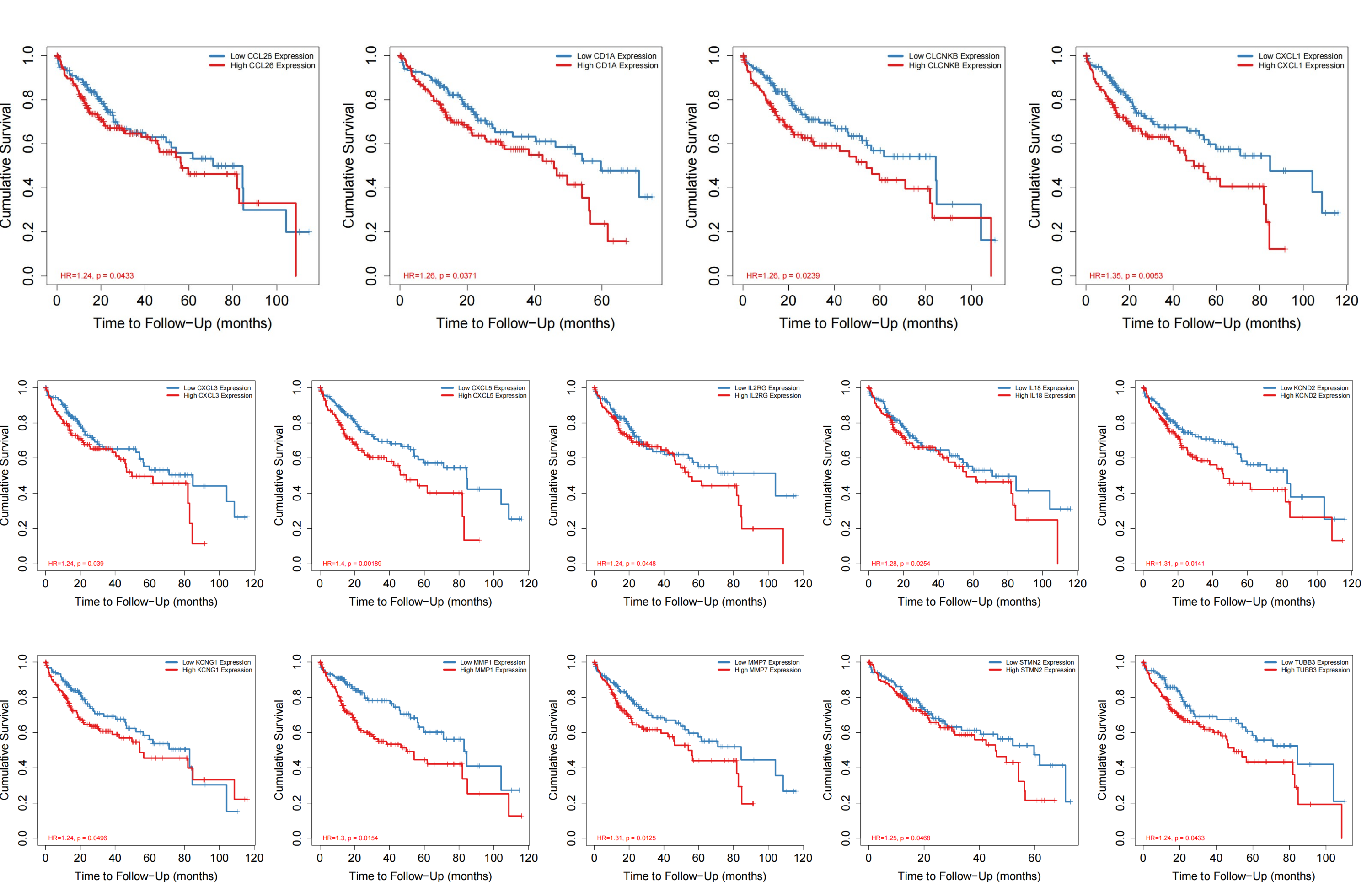

Supplement: Supplementary file 4 — Supplementary Figure S2. [file 41598_2023_34519_MOESM4_ESM.png]

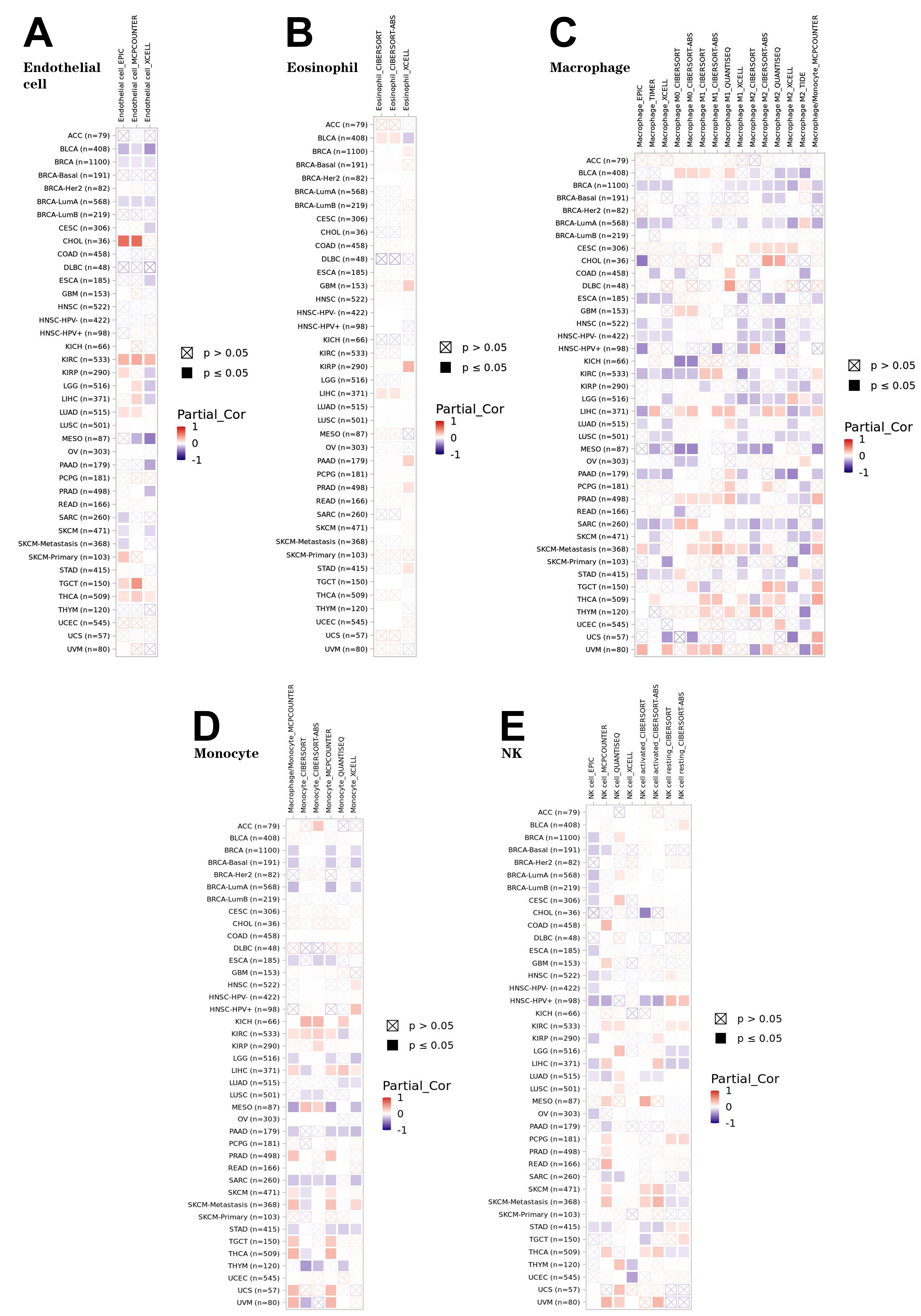

Supplement: Supplementary file 5 — Supplementary Figure S4. [file 41598_2023_34519_MOESM5_ESM.jpg]
